# EHMTI-0011. Spinal manipulation for a child with chronic cervicogenic headaches: a case report

**DOI:** 10.1186/1129-2377-15-S1-C2

**Published:** 2014-09-18

**Authors:** J Alcantara, R Olsen

**Affiliations:** 1 The International Chiropractic Pediatric Association, San Francisco, USA

## Background

Headaches are common in childhood and increases in frequency towards adolescence. Headache prevalence range from 37% to 51% in those ≤ 7 years of age and increases to 57% to 82% by age 15 years. Prior to puberty, boys are affected more frequently than girls, but following the onset of puberty, headaches occur more frequently in girls. Many sufferers turn to alternative therapies to augment their medical care. In the interest of evidence-informed practice, we describe the chiropractic care of a child with chronic cervicogenic headaches.

## Clinical features

A 6-year-old male with chronic headaches of 2 years duration presented for chiropractic care. No organic cause was determined by extensive medical diagnostics. Medical care consisted of ibuprofen which provided only minor relief.

## Intervention and outcome

The child was cared for with chiropractic care characterized as high velocity, low amplitude thrust-type spinal manipulation directed to sights of segmental dysfunction in the cervical spine. The patient attended care for a total of 10 visits over a 2-month period with resolution of the patient’s headache complaints.

## Conclusion

This case report provides supporting evidence in the use of spinal manipulation in the care of children with cervicogenic headaches.

No conflict of interest.

